# Preclinical cerebral cryoablation in non-tumor bearing pigs

**DOI:** 10.1038/s41598-022-05889-2

**Published:** 2022-02-07

**Authors:** Irena Jankovic, Frantz Rom Poulsen, Christian Bonde Pedersen, Bjarne Winther Kristensen, Tine Schytte, Thomas Lund Andersen, Louise Langhorn, Ole Graumann, Willy Krone, Poul Flemming Høilund-Carlsen, Bo Halle

**Affiliations:** 1 Department of Neurosurgery, Odense University Hospital, J. B. Winsløws Vej 4, 5000 Odense C, Denmark; 2 Department of Pathology, Odense University Hospital, Odense, Denmark; 3 Department of Oncology, Odense University Hospital, Odense, Denmark; 4 Department Nuclear Medicine, Odense University Hospital, Odense, Denmark; 5 Department of Nuclear Medicine, Odense University Hospital, Odense, Denmark; 6 Biomedical Laboratory, University of Southern Denmark, Odense, Denmark; 7 Department of Radiology, Odense University Hospital, Odense, Denmark; 8 Department of Clinical Research and BRIDGE (Brain Research-Inter Disciplinary Guided Excellence), University of Southern Denmark, Odense, Denmark

**Keywords:** Surgical oncology, CNS cancer

## Abstract

Patients with brain metastases, the most common intracranial tumor, have an average survival ranging from a few months to 40 months, and new treatment initiatives are needed. Cryoablation is a minimally invasive, well-tolerated, and effective procedure commonly applied for treatment of renal tumors and certain other malignancies. We aimed to examine the clinical usefulness of this procedure in a step-by-step program starting with cerebral cryoablation in healthy pigs. In four terminal and four non-terminal non-tumor bearing pigs, we studied immediate and delayed effects of cerebral cryoablation. Safety was assessed by computed tomography (CT), and clinical observation of behavior, neurological deficits, and wellbeing. Effects were assessed by histological and immuno-histochemical analyses addressing structural and metabolic changes supported by additional magnetic resonance imaging (MRI) and positron emission tomography (PET) in the non-terminal animals. Using CT-guidance, cryoablation probes were successfully inserted without complications, and ice formation could be monitored real-time with CT. No animal developed neurological deficits or signs of discomfort. Histological and immunohistochemical analyses, MRI, and PET revealed profound structural and biological damage within the lesion. MRI and PET revealed no long-term damage to healthy tissue outside the cryoablation zone. Cerebral cryoablation appears to be a feasible, safe, and controllable procedure that can be monitored successfully with CT. The net effect is a dead brain lesion without damage of either nearby or remote healthy structures. Immediate changes are local hemorrhage and edema; delayed effects are perfusion defects, immune system activation, and astrogliosis.

## Introduction

Brain metastases are the most common form of cerebral malignancy and can arise from a wide variety of primary tumors. Patients may have had several different treatments for their primary cancer, and resistance to multiple lines of therapy is common. Median survival varies widely and ranges from 7–47 months in non-small-cell lung cancer, 3–36 months in breast cancer, 5–34 months in melanoma, 3–17 months in gastrointestinal cancer, and 4–35 months in renal cancer^1^.

Length of survival is influenced by multiple factors including age, physical status, cancer subtype, and choice of treatment including surgery, stereotactic radiosurgery, whole-brain radiation therapy, chemotherapy, molecularly targeted therapeutics, and immunotherapies^1^. Cryoablation is a procedure in which a cryoprobe is used to freeze and destroy abnormal tissue; this approach has been successfully applied in a variety of malignancies including renal cancer and primary and secondary hepatic cancers^2,3^, but not yet for the treatment of secondary cerebral malignancies. Although cryoablation is a minimal invasive procedure, complications such as edema and hemorrhages are seen; in the brain, these could lead to neurological deficits^4,5^.

In a step-by-step program, we aim to examine the applicability of cryoablation in brain metastasis. In the first step, we investigated the method’s feasibility, safety, and efficacy in healthy pigs, which is what we report in the present article.

## Results

**Figure 1:**
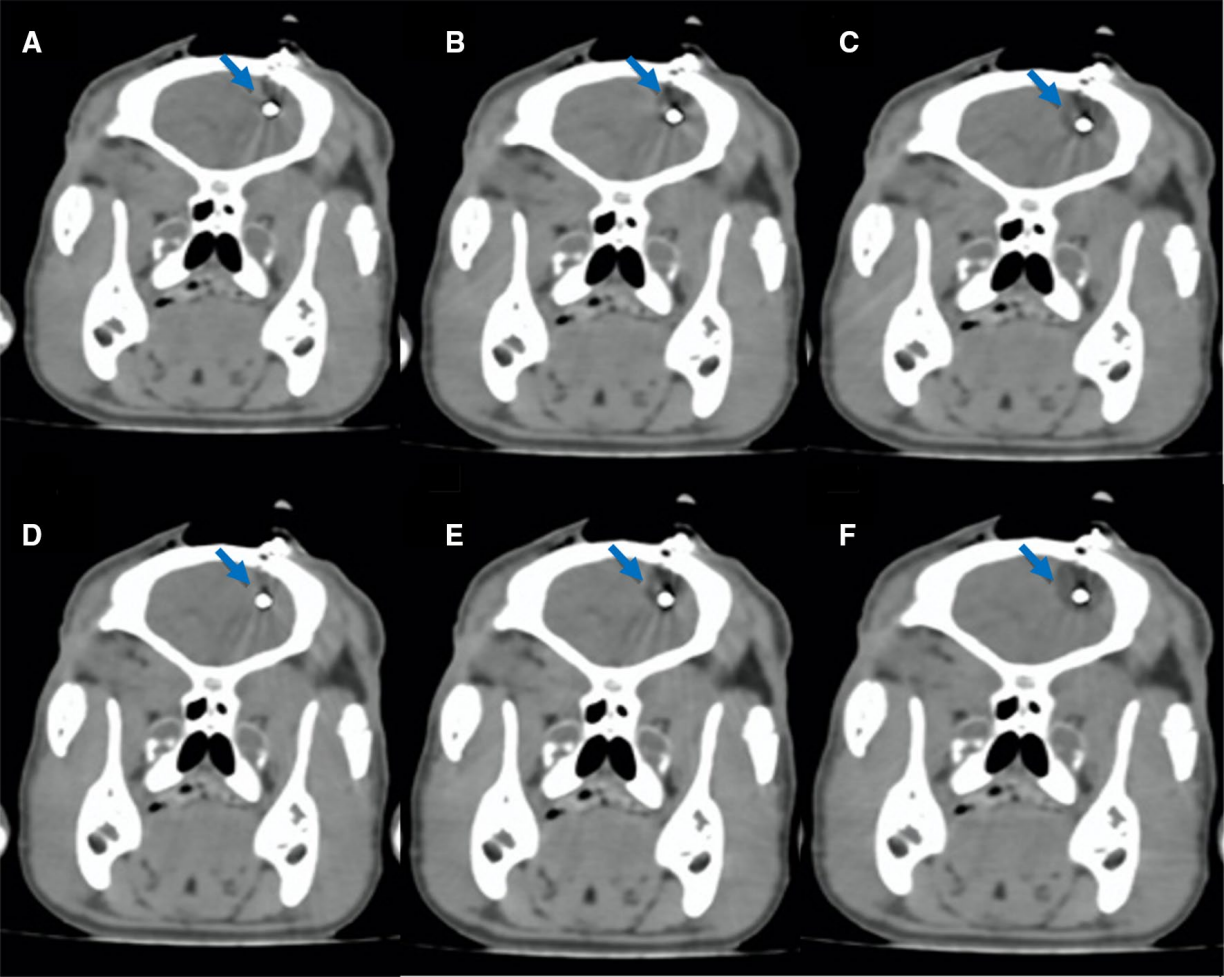
Metal artefact reduced coronal section CT scans of the brain after cryoablation (blue arrow). (**A**–**C**) Ice formation during the first freeze cycle; (**A**) after 0.5 min, (**B**) after 1.5 min, and (**C**) after 3 min. (**D**–**F**) Ice formation during the second freeze cycle; (**D**) Lesion size after 0.5 min, (**E**) after 1.5 min, and (**F**) after 3 min. No artefact caused by the probe was seen after metal artefact reduction.

**Figure 2:**
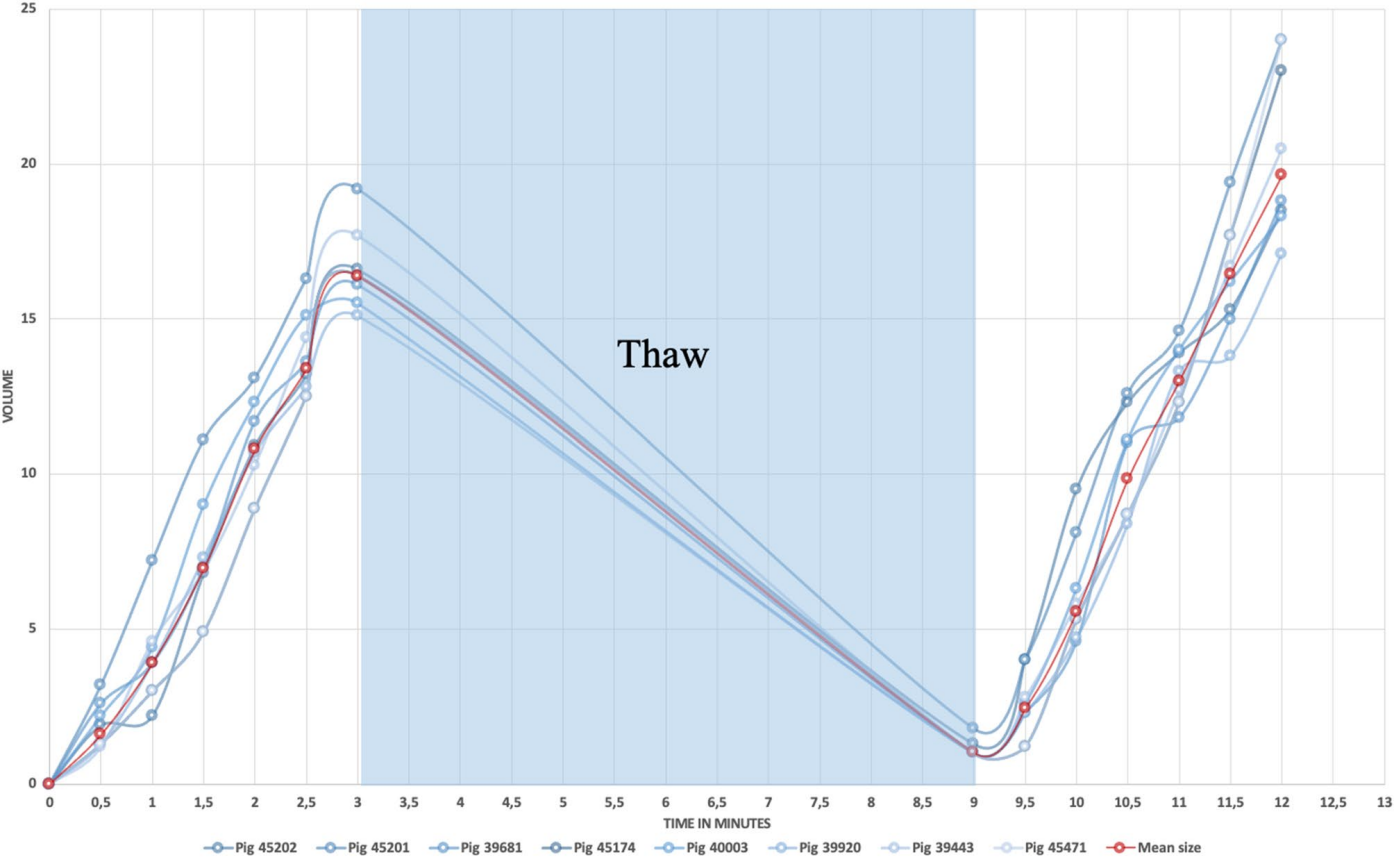
Ice volume development in cm^3^ as a function of time in minutes, shown for all 8 pigs. The red line represents mean values. The volume decrease in the thaw period is presumed as no CT scans were obtained in this phase.

The CT-guided cryotherapy was successfully performed in all eight animals. The stereotactic insertion of the cryoprobe into the right parietal lobe was completed without complications. Two of the pigs developed pneumocephalus, which did not affect the procedure. As expected, the cryoprobe caused metal artefacts on the CT scans. Despite this, it was possible to successfully follow the ice development during the procedure (Fig. 1). The size of the ice formation increased at the same rate in all pigs (Fig. 2).

The biggest increase in volume of the ice formation occurred after repetition of the freezing cycle, i.e. from 16.4 ± 1.3 cm^3^ to 19.7 ± 2.3 cm^3^, corresponding to a 20% increase in ice formation upon repeated freezing. The greatest difference in ice volume observed among pigs was 6.1 cm^3^ on CT. The observed mean volume on the MRI scans 13 days post procedure was 19.4 ± 3.7 cm^3^ and macroscopically 20.5 ± 3.5 cm^3^ (Table 1). The non-terminal group, which was clinically observed for 13 days, did not show any signs of behavioral changes or neurological deficits. No infection or wound healing problems were observed.

**Table 1:** Ice formation mean as a function of time.

Time (min)	Mean size (cm^3^)	SD (cm^3^)
0	0	0
0.5	1.6	0.7
1	3.9	1.5
1.5	7	2
2	10.8	1.5
2.5	13.4	1.4
3	16.4	1.3
9	1	0.3
9.5	2.5	1.1
10	5.6	1.7
10.5	9.9	1.8
11	13	1
11.5	16.5	1.8
12	19.7	2.8

### Pathological study

#### Gross examination

The mean diameter of the immediate lesions was 2.1 ± 0.22 cm. In the delayed lesions, the mean diameter was 1.7 ± 0.05 cm. The lesions were all macroscopically well-defined with a clear border to the non-frozen brain tissue.

#### Histology

The immediate effects seen on the hematoxylin–eosin (HE) staining showed bleeding and edema corresponding to the cryolesion. Some scattered macrophages were also seen. The delayed effects seen on the HE staining showed signs of bleeding in the periphery of the lesion. In the center of the lesion, pale necrotic-looking tissue was seen with characteristic paleness of the nuclei, cell bodies, and fibers. In the outer zone of the lesion, numerous macrophages were present. Neutrophils and lymphocytes were also present, but to a lesser degree. We used the contralateral side of the brain as a control, see Supplementary Fig. 1.

#### Immunohistochemical study

**Figure 3:**
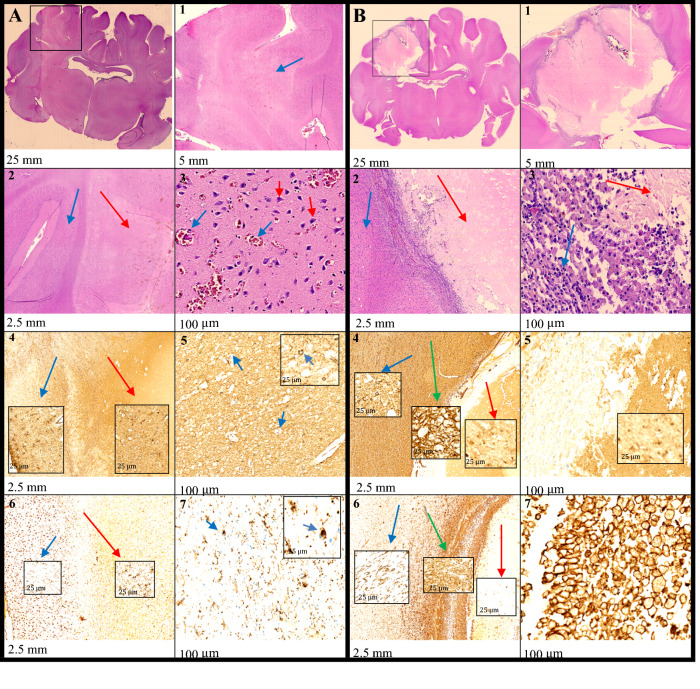
Histological and immunohistochemical analysis of (**A**) immediate and (**B**) delayed lesions. The square in the upper left corner of (**A**) and (**B**) marks the position of the lesion. The specimens have different staining and magnification. (**A 1**–**3**) HE staining, showing (**1**) the lesion (blue arrow); (**2**) border zone between normal tissue (blue arrow) and lesion (red arrow), and (**3**) hemorrhage (blue arrows) and neurons (red arrows) in the lesion center. (**A 4**–**5**) GFAP staining, demonstrating (**4**) border zone with normal tissue to the left (blue arrow) and lesion to the right (red arrow), where astrocytes have lost their normal star shape; (**5**) the GFAP staining of the lesions showing swelling of astrocytic cell bodies with loss of astrocytic stellate processes (blue arrows). (**A 6**–**7**) Iba1 staining, showing (**6**) border zone with normal tissue to the left (blue arrow) and lesion to the right (red arrow) and (**7**) microglial cells with swollen cell bodies and cell processes in the lesion center (blue arrows). (**B 1**–**3**) HE staining, showing (**1**) a pale and necrotic lesion with the border zone filled with cells and a small hemorrhage, (**2**) border zone between the normal tissue (blue arrow) and the lesion (red arrow), and (**3**) macrophages, lymphocytes, and neutrophils to the left (blue arrow) and necrotic lesion to the right (red arrow). (**B 4**–**5**) GFAP staining, demonstrating (**4**) border zone with normal tissue to the left (blue arrow) and lesion to the right (red arrow). Astrogliosis is seen towards the lesion (green arrow), whereas (**5**) the center of the lesion is without vital astrocytes. (**B 6**–**7**) Iba1 staining, showing (**6**) border zone with normal tissue to the left (blue arrow) and lesion to the right (red arrow) with an increased number of macrophages in the lesion periphery (green arrow), and (**7**) especially in the border zone.

The immediate effects seen on the binding adaptor molecule 1 (IBA1) staining were scattered macrophages as well as microglial cells with swollen cell bodies and cell processes. The glial fibrillary acidic protein (GFAP) staining showed astrocytes with round, swollen cell bodies and loss of astrocytic processes. The delayed effect on IBA1 staining was an increased number of macrophages especially in the border zone, but also in the lesion center. GFAP staining showed astrogliosis in the surrounding brain parenchyma and in the periphery of the lesion (Fig. 3). We used the contralateral side of the brain as a control, see Supplementary Fig. 1.

### MRI

**Figure 4:**
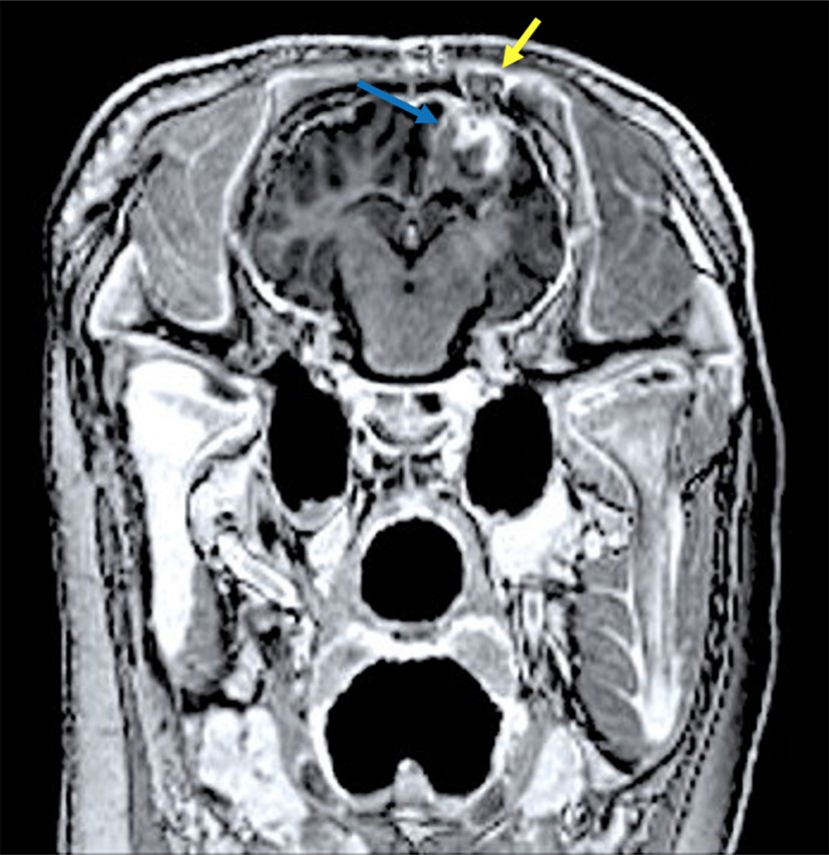
Postoperative T1 weighted MRI with contrast inversion prepared gradient echo coronal section of the brain and cryolesion. A large, well-defined lesion (blue arrow) is seen cranial to the right ventricle with a thin contrast enhancement peripherally. From the burr hole and into the cryolesion, a wedge-shaped lesion (yellow arrow) is seen, which is compatible with the spongostan used to close after the procedure.

The MRI scans showed expected postoperative changes following the frontoparietal burr hole to the right of the midline, with discrete thickening and increased contrast enhancement in the dura underlying the burr hole. Cranial to the right lateral ventricle was (as intended) a large, well-defined lesion that was hyperintense and slightly inhomogeneous on the FLAIR sequence with a discrete hypointense ring; isointense to white matter and slightly inhomogeneous on T1w sequences with discrete hyperintense signal and thin peripheral contrast enhancement on T1w contrast sequence. There were no perilesional signal changes. Underlying the burr hole, a wedge-shaped lesion was seen that was hypointense on T2w FLAIR sequences; isointense to white substance on T1w sequences with discrete contrast enhancement in the periphery—compatible with spongostan (absorbable hemostatic gelatin sponge) placed in the burr hole for closure at the end of the cryo-procedure (Fig. 4).

### PET

Static FDG-PET 30–45 min post injection and simultaneous MRI were performed 13 days after the cryo-ablation procedure. A manual co-registration to the initial CT demonstrated in all four pigs a relatively well-defined, near-spherical metabolic defect symmetrically surrounding the position of the freezing probe with profound and almost homogenously reduced FDG activity when compared to a corresponding reference region in the contralateral cerebral hemisphere. Maximal and mean standardized uptake values (SUVmax and SUVmean) were reduced by at least 50–60% and 25–45%, respectively.

**Figure 5:**
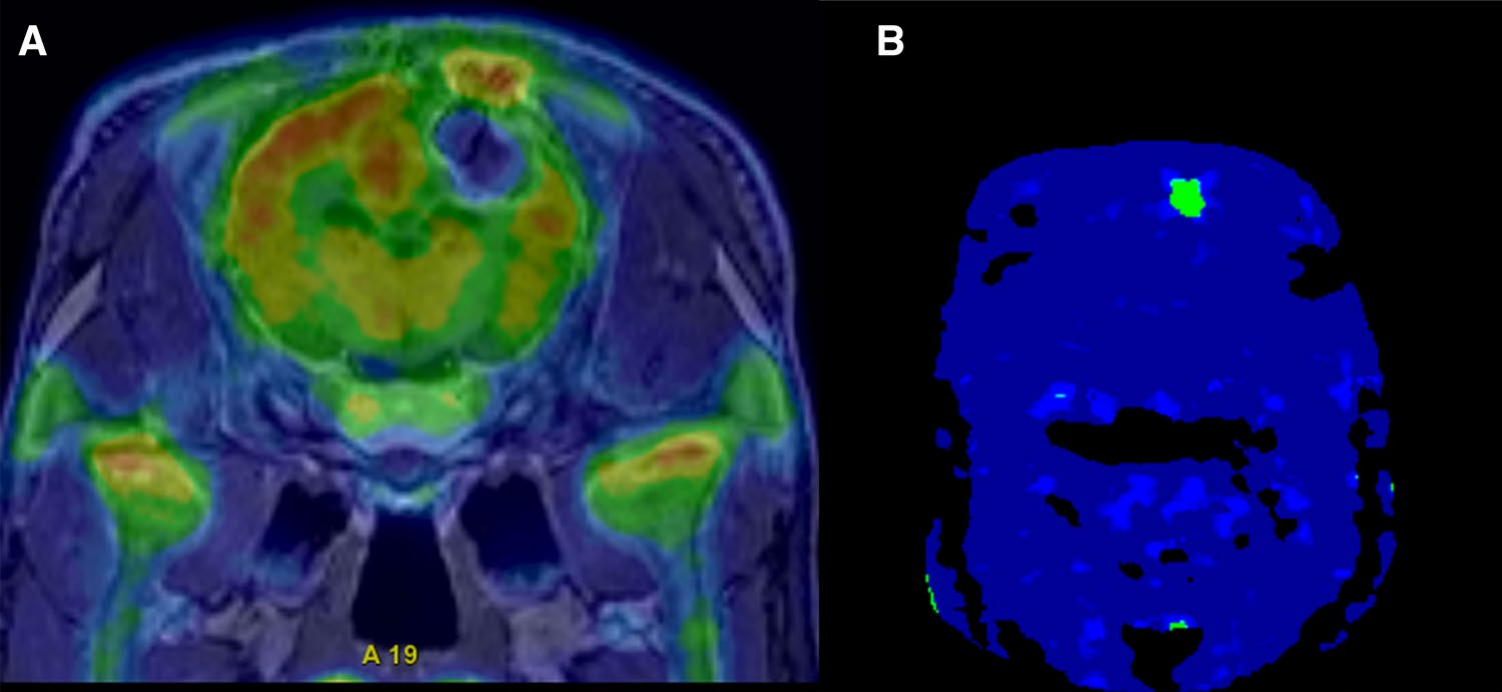
Postoperative static FDG-PET scan acquired 30–45 min after injection. (**A**) Relatively well-defined near-spherical metabolic defect symmetrically surrounding the position of the freezing probe showing profoundly and almost homogenously reduced FDG activity (light blue arrow) compared to the same area in the contralateral hemisphere (dotted white oval). (**B)** PET compartment of dynamic FDG scan showing enhanced k_3_ signal, indicating increased activity (yellow arrow).

In the margin of the central main part of the lesion, a narrow rim with higher FDG activity was observed. In all four pigs, the FDG findings closely matched the MRI T2w FLAIR signal, with a high signal in the central metabolic effect and a low signal in the rim. On the T1w contrast sequences, the rim showed a high degree of contrast enhancement. PET compartment modeling of dynamic FDG scans showed enhanced signal in the k_3_ phase (Fig. 5).

## Discussion

We found that CT-guided cerebral cryoablation was feasible. The cryoablation procedure was performed without problems and with perfect CT-guidance in all pigs. The ice formation was easily monitored despite the metal artefacts on the scans caused by the cryoprobe. No pigs showed signs of neurological deficits after the procedure. The immediate effects seen on histological and immunohistochemical analysis were edema and bleeding as well as cell destruction. The delayed histological and immunohistochemical effects were an increased number of immune cells and astrogliosis. The stereotactic placement of the probe was technically simple and safe. The standard Stealth™ Navigus™ skull-mounted trajectory kit was used with similar set up as for stereotactic frameless biopsy. The ice’s greatest volume increased by 20% with a second freezing cycle. This corresponds well to the theory that using two freezing cycles enhances the effect^4,7^. The greatest observed difference in ice volume was 6.1 cm^3^, which is acceptable. The mean volume seen on the MRI scans 13 days post procedure was 19.4 ± 3.7 cm^3^ and macroscopically 20.5 ± 3.5 cm^3^. The lesion size did not change much from the initial CT scan to the final MRI undertaken 13 days after the procedure. Likewise, the lesion size did not vary between CT, MRI, and histology.

Effects of cryoablation are both direct and indirect. Direct cell damage is a result of ice crystal formation, first extracellular (0 to − 20 °C), leading to intracellular dehydration caused by an osmotic shift, and then intracellular (< − 20 °C)^8,9^. Indirect cell damage is due to an osmotic shift causing smaller vessels to swell, which leads to endothelial damage that causes edema and, most importantly, thrombosis^6,7^. Thrombosis in small vessels is seen after 3–4 h and in larger arteries after 1 day, resulting in hypoxia and cell death^6,8^. The indirect cell damage corresponds to the PET findings, i.e. a near-spherical metabolic defect with homogenously reduced FDG activity compared to the contralateral cerebral hemisphere. SUVmean was reduced by 25–45%. This could indicate a perfusion defect in relation to the metabolic defect, which inhibits FDG uptake within the lesion. However, increased enzymatic activity was seen in the k_3_ phase, indicating that there were a few surviving neurons left within the lesion capable of taking up FDG. To our knowledge, no prior study has used PET to evaluate the metabolomics within a cryolesion. The PET scans further revealed an increased FDG uptake in the periphery of the lesion, indicating increased metabolic activity. Histologically, an increased number of leucocytes and microglial cells were observed in the periphery of the lesion on the HE and Iba1 staining. On the GFAP staining, astrogliosis was also seen in the periphery. This corresponds well to the hyperintense signal seen on PET. We find it likely that this indicates a local immune response in the brain induced by the cryoablation.

James Arnott first used cryotherapy to treat cervical uterine cancer in 1850^10^. A good hundred years later, Cooper and associates developed the first closed cryotherapy system and coined the term cryoablation, and modern cryotherapy was born^8,11^. The development in diagnostic imaging, especially CT and MRI, has made it possible to monitor the ice formation during the procedure, thereby increasing the safety and accuracy of cryoablation^12^. However, despite over 150 years of development, cryoablation has still not been implemented as a treatment option for brain metastases.

While CT is used for monitoring cryoablation of renal cancers^13,14^, MRI has been used for monitoring cryoablation in the brain^6,9^. Other ablation modalities, e.g., laser interstitial thermal therapy, use MRI for monitoring the procedure and placement of the probe^6,15^. We used CT scans for probe placement and monitoring as compatible cryoablation equipment was already used and present at our institution. Implementing cryoablation for the treatment of brain metastasis requires access to intraoperative CT or MRI, which unfortunately is often not the case despite the fact that CT- and MRI-guided cryoablation may well be clinically relevant.

Other studies have shown an increased immune response after extracerebral cryoablation, a so-called cryo-immunologic response. Soanes et al. were some of the first to report the presence of an immune response after cryoablation^16^. They observed antibodies specific to the prostate and a remission in metastatic disease^16^. Whether there is a beneficial antitumor immune response correlated to cryoablation is still not clear, but many animal studies suggest this^17–19^. The MRI T1w sequence with contrast showed contrast enhancement in the periphery of the lesion, which indicates a fragmented blood–brain-barrier in line with the presumed endothelial damage leading to indirect cell damage.

The typical complications associated with cryoablation are edema, hemorrhage, and new neurological deficits^5,8^. These complications are typically seen immediately after cryoablation or within the first couple of days^6^. In a Russian study, 2.3% of tumor patients treated with cryoablation developed edema, and 4.5% developed hemorrhages. A neurological morbidity rate of 42% was reported, which is comparable to surgical resection of tumors in functionally important areas of the brain. In 8% of the cases, the loss of neurological function was permanent^5^. In a Canadian study, the overall complication rate was 62% after tumor-related craniotomy^20^, while the complication rate was 40% following stereotactic radiotherapy in an American brain tumor study^21^. In our study, no signs of symptomatic edema, hemorrhage, new neurological deficits, or behavioral changes were observed, and no wound infections occurred, all of which indicate a safe procedure.

### Limitations

It was not possible to obtain valid temperature measurements around the cryoprobes due to a lack of appropriate equipment, which meant that the lethal temperature could not be assigned. This could have helped us determine a precise lethal temperature for cryoablation. For the future, it would be advantageous to develop a dual probe with an incorporated thermometer in the cryoprobe. The development in lesion size after 2–3 days or at 7 days following the procedure was not recorded. Thus, we were not able to follow the natural course of lesion development in the days after the procedure. Pig 45,471 differed from the rest on MRI, possibly due to individual variation and measurement uncertainty. The outliers in the CT scans were due to pig 39,920, which on the day of cryoablation had fever (41 °C) that presumably caused less ice formation, and pig 45,471, which during the cryoablation developed hypothermia that presumably caused the large ice formation. This indicates a need for whole body temperature monitoring during the procedure for a more predictable outcome. Finally, our study was preclinical and conducted in non-tumor bearing pigs, and the study sample size was small, so conclusions should be made with caution. However, if additional studies in tumor-bearing pigs show similarly positive results with regard to safety and efficacy, cryoablation might become a valuable adjunct to modern surgical and radiotherapy management of brain metastases. Brain metastases pose a problem when there are several of them and they are in hard-to-reach areas of the brain. It is not yet known how many metastases can be treated at a time with cryoablation. It is assumed that guidelines for surgical removal and stereotactic radiation therapy are also applicable to cryoablation^1,2^, but studies focusing on this are needed before conclusions can be made.

## Materials and methods

### Animal model

The study was conducted on eight female Danish Landrace pigs weighing approximately 20 kg. The pigs were divided into two subgroups, a terminal group and a non-terminal group, to evaluate the immediate and delayed effects of cerebral cryoablation. The pigs arrived at the research housing facility 1 week prior to the procedure to ensure proper acclimatization. All procedures were performed with the animals under general anesthesia. This was induced by a Zoletil mixture consisting of 250 mg Tiletamine + Zolazepam, 6.25 ml Xylazine 20 mg/ml, 1.25 ml Ketamine 100 mg/ml, 2 ml Butorphanol 10 mg/ml, and 2 ml Methadone 10 mg/ml. The dose was 1 ml per 10 kg. The mixture was administered intravenously (i.v.) and supplemented every 30 min either i.v. or intramuscularly, and all pigs were given supplementary oxygen to maintain normal saturation.

The operative procedure was started by making a 10 cm midline incision at the top of the skull to expose the frontal and parietal bones, and a 3 mm long titanium screw was placed in bregma to be used as a fix-point. The pigs were then placed in prone position on a CT table, and a CT scan of the head was made. This was used to determine the exact placement of a 10 mm wide burr hole on the right side to allow stereotactic insertion of a cryoprobe into the parietal lobe. In the majority of cases, this was 1.5 cm lateral to the inserted guide screw. After the burr hole was made, the dura was incised and a Stealth™ Navigus™ skull-mounted trajectory kit (Medtronic, Copenhagen, Denmark) normally used for frameless biopsy in the human brain was fixated over the burr hole. Under CT guidance, the cryoprobe was inserted through the trajectory kit to a depth of 1.5–2 cm lateral to the ventricular system in the right parietal lobe. The cryoablation was then started. The freeze–thaw-freeze cycle was 3 min–6 min–3 min^6^. During the freeze cycles, the ice formation was monitored every 30 s with consecutive CT scans. Following cryoablation, the terminal group was kept under general anesthesia for 2 h before euthanasia. The non-terminal group was awakened and clinically observed in the animal housing facility for 13 days (twice a day for the first four days and then once a day). After the 13 days of clinical observation, the pigs were scanned with positron emission tomography/magnetic resonance imaging (PET/MRI) before being euthanized. The pig brains were then surgically removed, fixated for 13 days in formalin, embedded in paraffin, and evaluated histologically.

### Cryoablation

The study was performed using a the Icefx™ Cryoablation System (Boston Scientific, Marlborough, Massachusetts, USA), and the cryoprobes used were IceSphere™ 1.5 CX 90° (BL Medical ApS, Jyllinge, Denmark).

### CT system and sequences

Pigs were CT scanned using a GE Discovery MI PET/CT scanner (GE Healthcare, Waukesha, USA). They were scanned in the rostral caudal direction with initial alignment of the pig performed by a localizer. Subsequent CT during freezing was performed every 30 s using a 120 kVp (peak kilovoltage) and 300 mA (milliampere) current in a helical trajectory covering the animal’s skull. To avoid metal artefacts from the probe and other metal objects in the field of view, a metal artefact reconstruction (MAR) with a slice thickness of 0.625 mm was made. Final images were in-plane reconstructed in 512 × 512 matrices in a 50 cm field of view.

### PET/MRI system and sequences

PET/MRI scans were conducted on a GE Signa 3 T PET/MRI (GE Healthcare, Waukesha, USA). Approximately 50 megaBecquerel (MBq) Flour-Deoxy-Glucose (FDG) was injected i.v. as a bolus into the pigs at which time a 45-min dynamic PET scan was started. During the PET scan, an attenuation correction scan based on MRI was initially acquired by a zero-echo time (ZTE) sequence with subsequent segmentation to a bone, air, and water attenuation map as implemented in software version MP26R03 on the Signa PET/MRI. A sagittal 3D T1w inversion prepared gradient echo sequence (BRAVO) was then acquired with an isotropic resolution of 0.8 × 0.8 × 0.8 mm^3^ using an echo time of 3.4 ms, a repetition time of 8.6 ms, a flip angle of 12°, and an inversion angle of 180°. Secondly, a sagittal 3D T2w FLAIR (fluid attenuated inversion recovery) was acquired with an isotropic resolution of 1 × 1 × 1 mm^3^ using an echo train length of 170, inversion time of 1727 ms, an echo time of 120 ms, and a repetition time of 6200 ms. After this, 1 ml of contrast agent Gadovist (1 mmol/mL) was injected through a peripheral vein, and after it had circulated for 1 min in the bloodstream, a 3D T1w scan with scan parameters as above was performed. PET images were reframed to a static scan 30–45 min post injection along with a 12 × 10 s–8 × 5 m–1 × 3 m dynamic framing reconstructed by a Bayesian penalized reconstruction method (Q.Clear) using a β-parameter of 500 with attenuation and scatter correction based on the pseudo-CT derived from the ZTE.

### Histology and immunohistochemistry

After fixation for 13 days in 10% neutral buffered formalin, the brains were sectioned in 0.5 cm thick coronal slices. The sections were grossly examined using a ruler. Specimens of the cryolesion, the surrounding tissue, and the untreated area were embedded in paraffin, followed by preparation of 2–3 $\mathrm{\mu m}$ thick histological sections, which were stained with hematoxylin–eosin (HE). Immunohistochemistry used antibodies targeting Iba1 to identify microglial cells and macrophages, and GFAP to identify astrocytes and potential astrogliosis. The HE staining was run on Dako CoverStainer using the following reagents: Dako Eosin (Ref: CS701), Dako Bluing Buffer (Ref: CS702), and Dako Hematoxylin (CS700). The immunohistochemical staining was performed using a BenchMark DISCOVERY-platform and for detection we used OptiView (Ref: 760–700 from Ventana), Hematoxylin II (Ref: 790–2208 from Ventana), and Bluing Reagent (Ref: 760–2037 from Ventana). The IBA1 antibody used was from Wako Pure Chemical Industries (code: 019-19741), and the protocol was 16 min of incubation with the primary antibody at 36 °C; demasking CC1 for 32 min at 100 °C. The dilution of primary antibodies was 1:2000 in Dako Antibody Diluent (Ref: S2022). The GFAP antibody used was from DAKO (code: Z033429-2), and the protocol was 24 min of incubation with the primary antibody at 36 °C; demasking CC1 for 32 min at 100 °C. The dilution of primary antibodies was 1:4000 in Dako Antibody Diluent (Ref: S2022).

### Tumor volume assessment

As described above, the fixated brains were cut in 0.5 cm thick coronal slices. On each slice, the radius a and b of the ellipsoid-shaped lesion was measured with a ruler. The area of the lesion was calculated using the formula, A = π*a*b, and the lesion volume on each slice was determined by multiplying by 4/3. The lesion volumes of all the slices were then summed to generate the total lesion volume.

The MRI scans were assessed and described by an experienced neuroradiologist using Picture Archiving and Communication Systems (PACS). The lesion was measured in cm in coronal, sagittal, and axial planes with the measuring feature embedded in PACS.

On the CT scans, cryoablation lesion sizes were measured in cm in coronal, sagittal, and axial planes using the measuring tools embedded in OsiriX Lite (Pixmeo SARL, Bernex, Switzerland). The volume was measured every 30 s throughout the ablation period. The volume was not measured during the thaw period.

For all three modalities, lesion volume was calculated by approximating the lesion to an ellipse and using the formula for ellipsoids, A = 4/3*π*a*b. The CT-calculated volume was then correlated to the volume measured in the histological specimens and the MRI lesions. Means and standard deviations were calculated and compared for the three data modalities.

### Data analysis

PET compartment modeling of dynamic FDG PET scans was performed using an image-derived arterial input function corrected for partial volume and background. Compartment modeling was calculated using a two-tissue model using first-order rate constants of K_1_, k_2_ and k_3_ where K_1_ [mL/cm^3^/min] is the rate constant from blood to tissue, k_2_ [1/min] the rate constant from tissue to blood and k_3_ [1/min] the rate constant to the binding compartment, i.e., the phosphorylation of FDG to FDG-6-phosphate. Calculations were performed on a voxel-by-voxel basis throughout the volume. Fits of time-activity curves was weighted by number of counts in the individual frames to improve the fit quality. From the fits K_1_, k_2_ and k_3_ were extracted. To evaluate the FDG uptake difference between the lesion and healthy brain tissue, signal intensity was obtained from an area within the lesion and compared to another within the corresponding brain parenchyma of the contralateral parietal lobe.

### Ethical approval

The study was approved by the national Animal Experiments Inspectorate. Case number 2019-15-0201-01649. All procedures performed involving animals were in accordance with the ethical standards of the institution at which the studies were conducted. This manuscript is reported in accordance with the ARIIVE guidelines.

## Conclusion

Results in healthy pigs indicate that cryoablation is a feasible procedure in the brain, and that ice formation can be monitored with CT during ablation. The method seems safe and effective, causing focal structural damage to produce a metabolically dead lesion without damage to nearby or remote cerebral structures. Immediate effects are local hemorrhage and edema, whereas delayed effects are perfusion defects, immune system activation, and astrogliosis. Studies of cryoablation in tumor-bearing pigs are needed before the procedure can be used in patients with brain metastases.

## Supplementary Information

Supplementary Figure S1.
