# Supplementary material for: Preclinical cerebral cryoablation in non-tumor bearing pigs
Source: Sci Rep. 2022 Feb 7;12:1977. doi: 10.1038/s41598-022-05889-2 (PMC8821607; doi:10.1038/s41598-022-05889-2)
Supplement: Supplementary file 1 — Supplementary Figure S1. [file 41598_2022_5889_MOESM1_ESM.docx]

Supplementary

**Figure s1.** As control in the histological and immunohistological examinations the contralateral hemisphere/side was used. Examples of immediate lesions are depicted in 1-6; HE staining in 1-2, GFAP staining in 3-4 and IBA1 staining in 5-6. Examples of delayed lesions are depicted in 7-12; HE staining in 7-8, GFAP staining in 9-10 and IBA1 staining in 11-12

| Contralateral side | Lesion side |
| --- | --- |
| 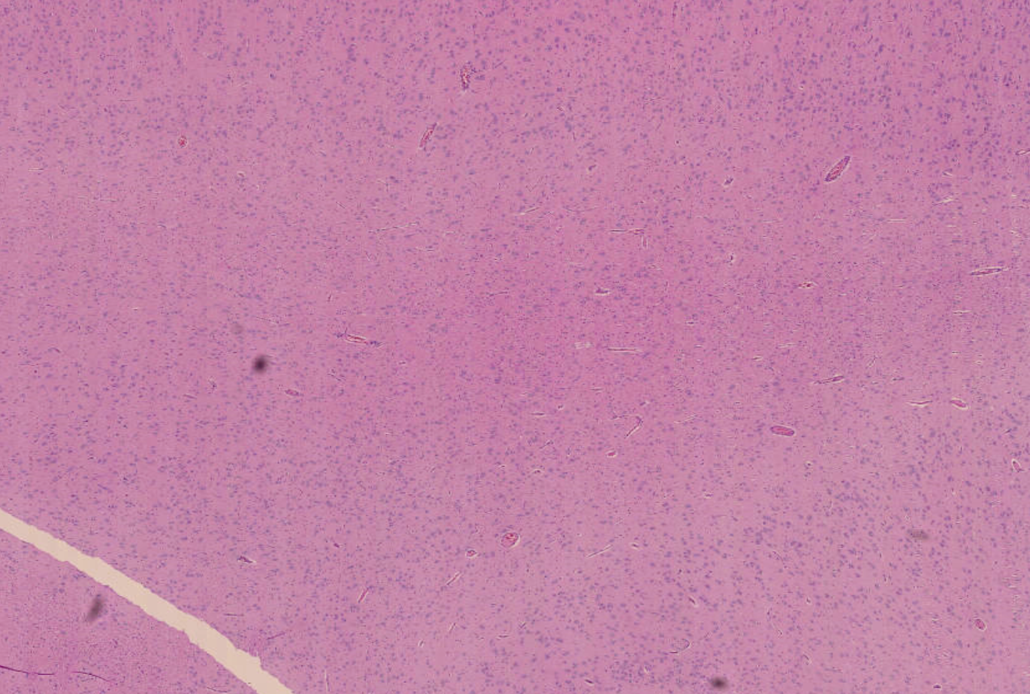  1 | 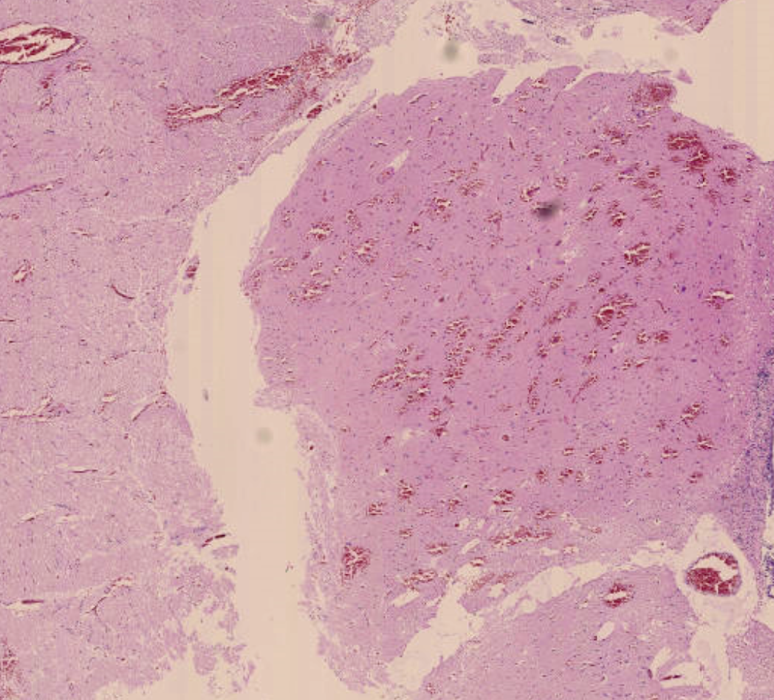  2 |
| 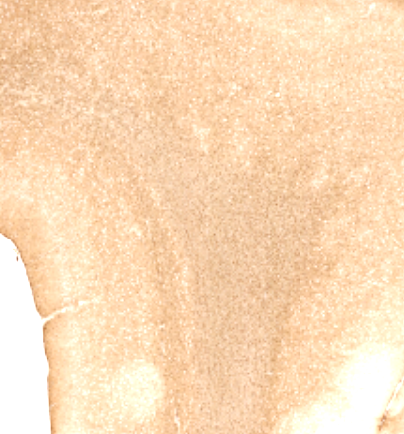  3 | 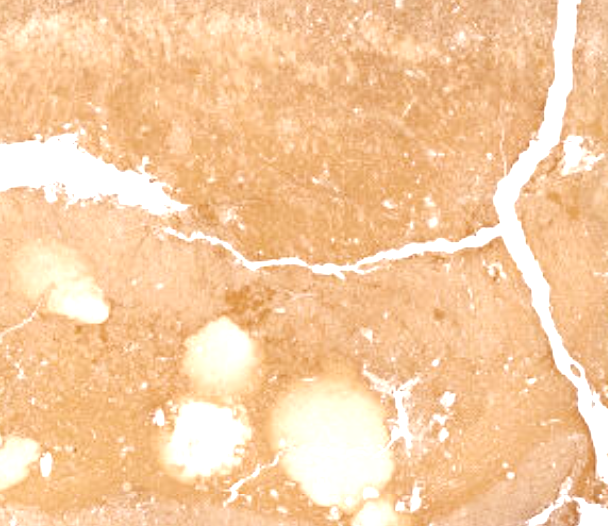  4 |
| 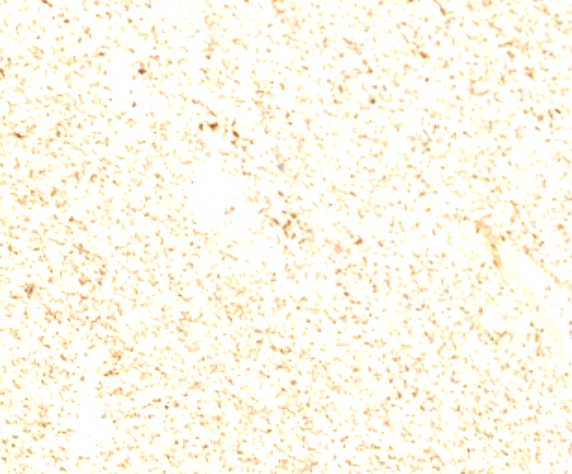  5 | 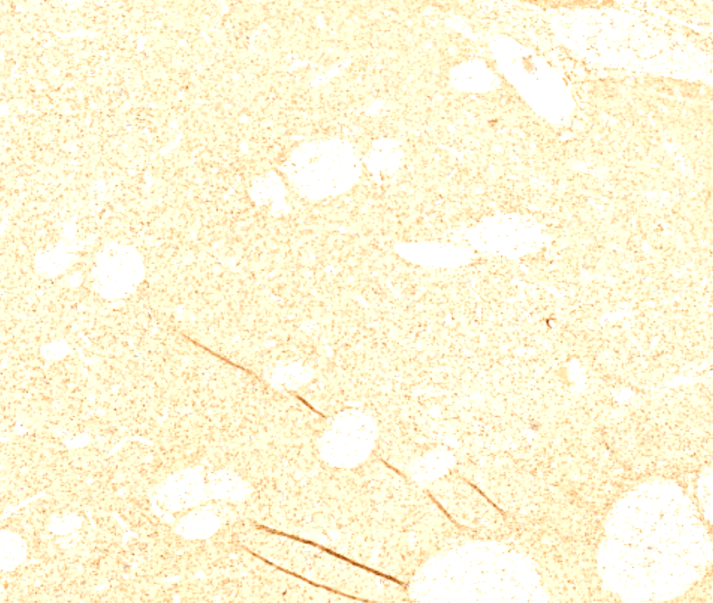  6 |
| 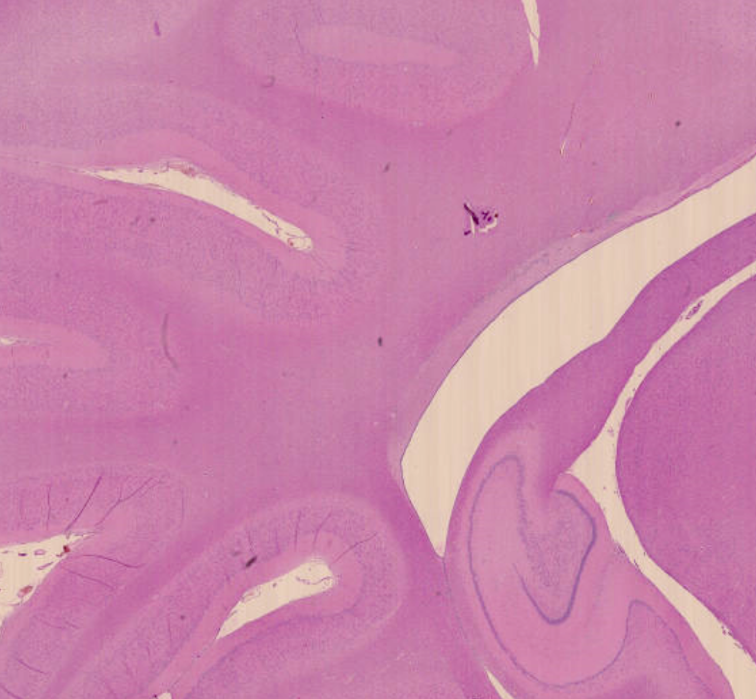  7 | 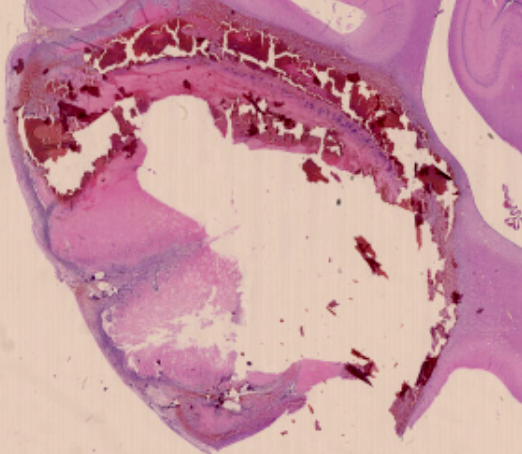  8 |
| 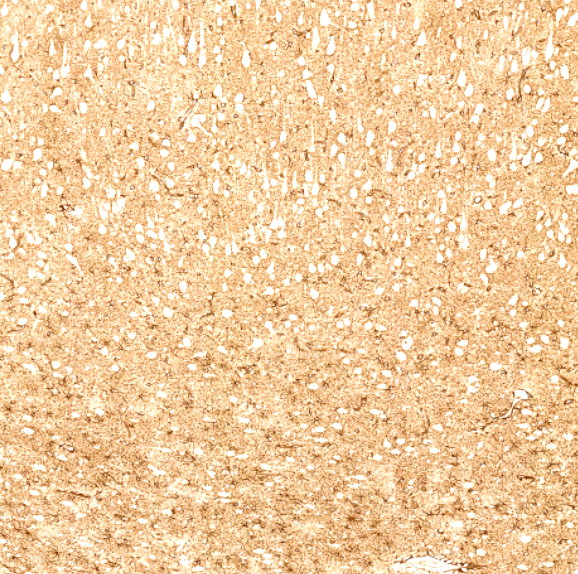  9 | 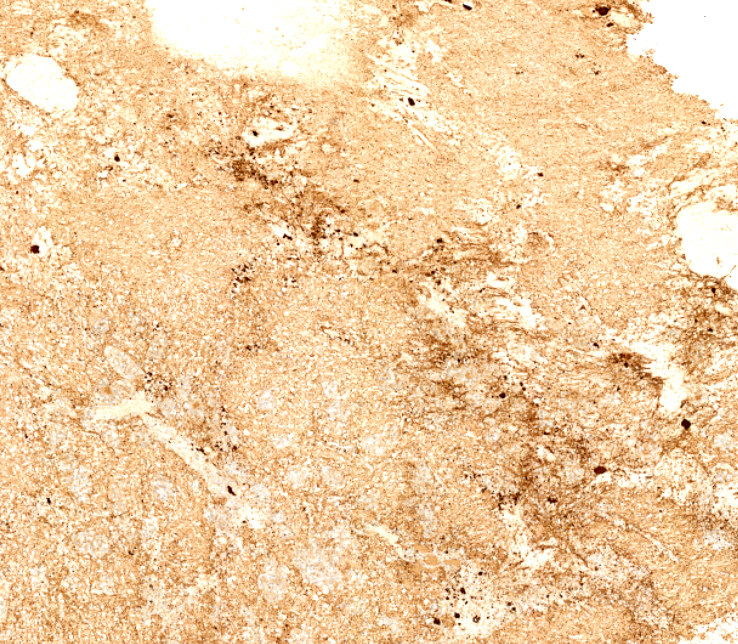  10 |
| 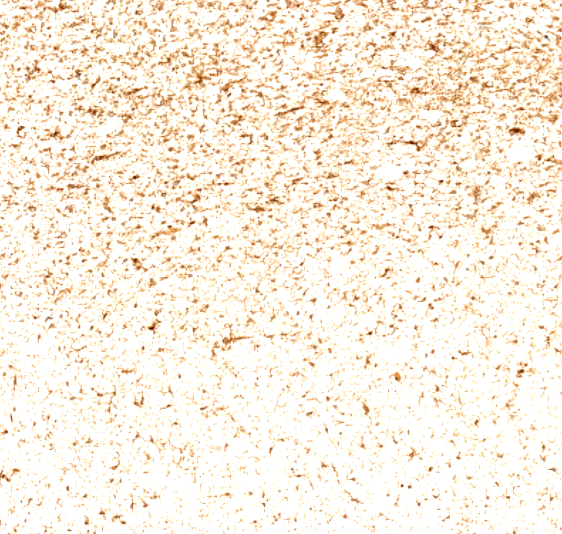 | 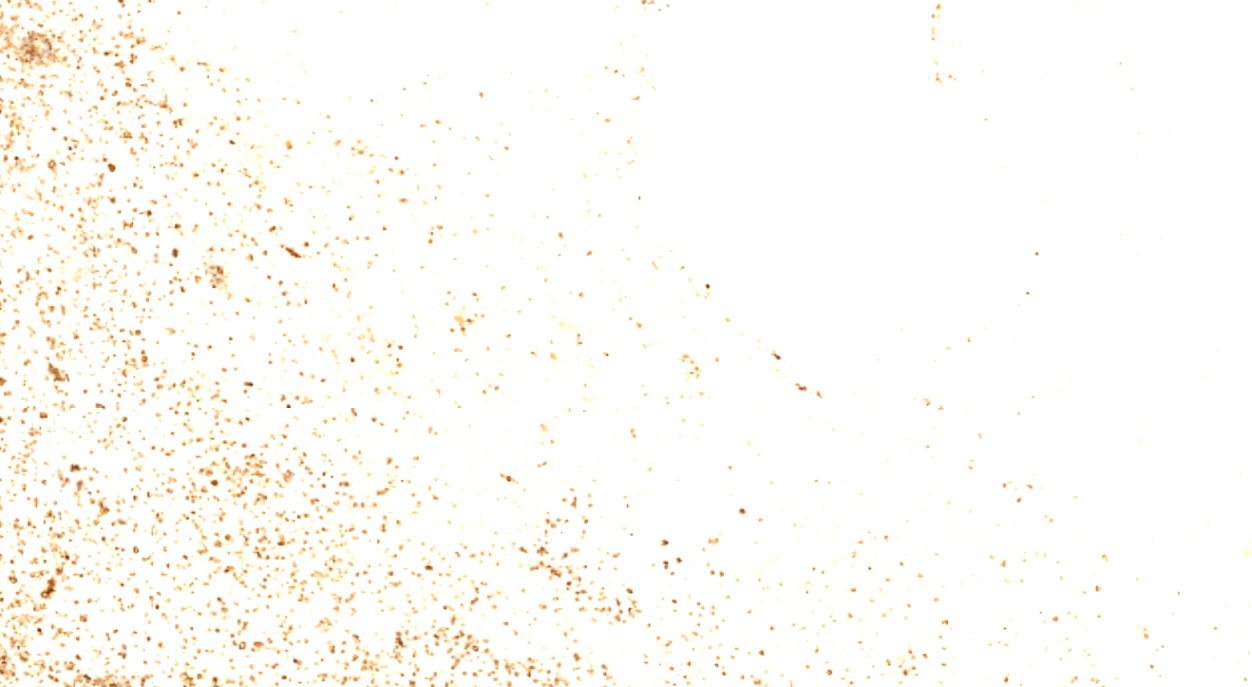 |

12

11
